# Supplementary material for: Possible itinerant excitations and quantum spin state transitions in the effective spin-1/2 triangular-lattice antiferromagnet Na2BaCo(PO4)2
Source: Nat Commun. 2020 Aug 24;11:4216. doi: 10.1038/s41467-020-18041-3 (PMC7445251; doi:10.1038/s41467-020-18041-3)
Supplement: Supplementary file 1 — Supplementary Information [file 41467_2020_18041_MOESM1_ESM.pdf]

Supplementary Information for

Possible itinerant gapless excitations and quantum spin state transitions in the effective spin-1/2 triangular-lattice antiferromagnet $Na_2BaCo(PO_4)_2$

N. Li[1,6], Q. Huang[2,6], X. Y. Yue[3,6], W. J. Chu[1], Q. Chen[2], E. S. Choi[4], X. Zhao[5], H. D. Zhou[2★], and X. F. Sun[1,3★]

[1]Department of Physics, Hefei National Laboratory for Physical Sciences at Microscale, and Key Laboratory of Strongly-Coupled Quantum Matter Physics (CAS), University of Science and Technology of China, Hefei, Anhui 230026, People's Republic of China

[2]Department of Physics and Astronomy, University of Tennessee, Knoxville, Tennessee 37996-1200, USA

[3]Institute of Physical Science and Information Technology, Anhui University, Hefei, Anhui 230601, People's Republic of China

[4]National High Magnetic Field Laboratory, Florida State University, Tallahassee, FL 32310-3706, USA

[5]School of Physical Sciences, University of Science and Technology of China, Hefei, Anhui 230026, People's Republic of China

[6]These authors contributed equally: N. Li, Q. Huang, X. Y. Yue.

[★]email: hzhou10@utk.edu; xfsun@ustc.edu.cn

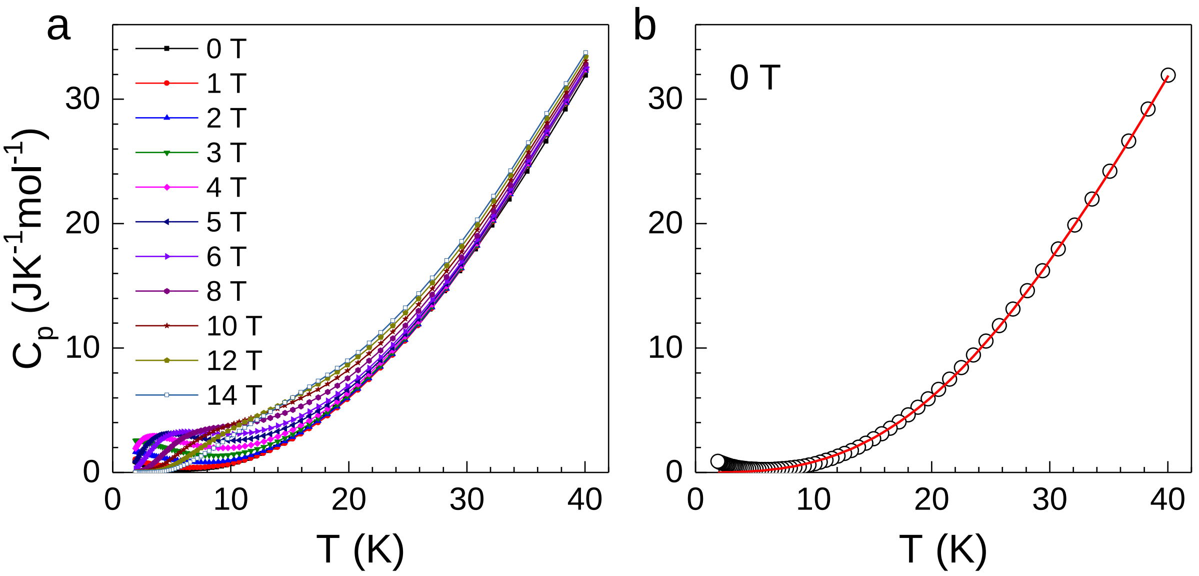

Supplementary Figure 1 **a**, Specific heat of $Na_2BaCo(PO_4)_2$ single crystal at 1.9 – 40 K and in various magnetic fields up to 14 T. **b**, Zero-field data. The solid line shows the fitting to data at $T >$ 10 K by using the formula of phonon specific heat, $C_{ph} = \beta T^3 + \beta_5 T^5 + \beta_7 T^7$.

Figure 1a shows the specific heat data of $Na_2BaCo(PO_4)_2$ single crystal at 1.9 – 40 K and in various magnetic fields of 0 – 14 T. These data are consistent with those reported in by Zhong *et al.*[1] There is no sign of phase transition in this temperature range. With applying magnetic field, a broad peak appears at low temperature. To estimate the phonon specific heat, we fit the zero-field data at 10 – 40 K by using the low-frequency expansion of the Debye function, $C_{ph} = \beta T^3 + \beta_5 T^5 + \beta_7 T^7$, where β, β_5, and β_7 are temperature-independent coefficients[2], as shown by the solid line in Fig. 1b. The fitting parameters are $\beta = 8.83 \times 10^{-4}$ JK^{-4}mol^{-1}, $\beta_5 = -3.32 \times 10^{-7}$ JK^{-6}mol^{-1}, and $\beta_7 = 6.67 \times 10^{-11}$ JK^{-8}mol^{-1}. Note that at very low temperatures, the T^5 and T^7 terms are negligible and the phonon specific heat shows a well-known T^3 dependence with the coefficient of β. As also mentioned in the main text, the phonon velocity can be calculated from the β value as v_{ph} = 2430 ms^{-1}, which is of a reasonable value for oxide materials. For a comparison, the phonon velocity of another QSL candidate, $Tb_2Ti_2O_7$, is 3440 ms^{-1} [3], determined in the same method.

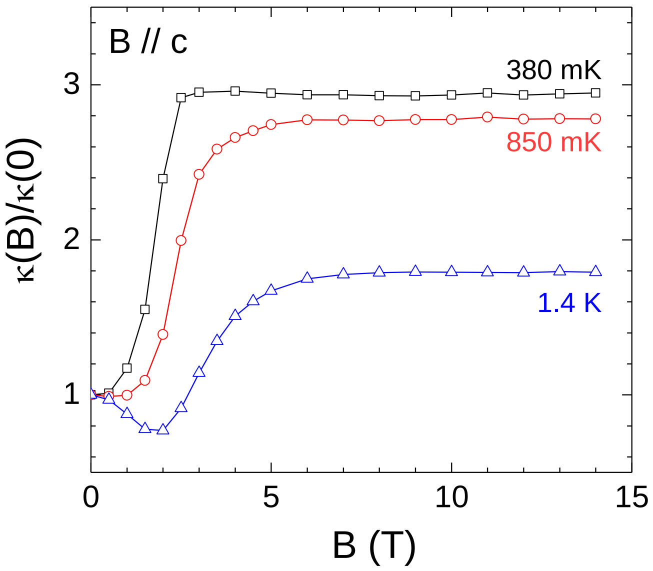

Supplementary Figure 2 Magnetic field dependence of thermal conductivity for $Na_2BaCo(PO_4)_2$ single crystal at different temperatures with *B* // *c*.

Figure 2 shows magnetic field dependence of thermal conductivity for $Na_2BaCo(PO_4)_2$ single crystal at 380 mK, 850mK and 1.4 K. At higher temperature, the $\kappa(B)$ shows a bit more different behavior, that is, with increasing field the κ first decreases at low field and then increases. This phenomenon is similar to that observed in some other magnetic systems, like $Yb_2Ti_2O_7$[4], and can be understood as the opposite roles of spin excitations as both carrying heat and scattering phonons.

SUPPLEMENTARY REFERENCES

1. Zhong, R. D. *et al*. Strong quantum fluctuations in a quantum spin liquid candidate with a Co-based triangular lattice. *Proc. Natl. Acad. Sci.* **116**, 14505-14510 (2019).
2. Tari, A. *Specific Heat of Matter at Low Temperatures Ch. 2* (Imperial College Press, London, 2003).
3. Li, Q. J. *et al.* Phonon-glass-like behavior of magnetic origin in single-crystal $Tb_2Ti_2O_7$. *Phys. Rev. B* **87**, 214408 (2013).
4. Tokiwa, Y. *et al.* Possible observation of highly itinerant quantum magnetic monopoles in the frustrated pyrochlore $Yb_2Ti_2O_7$. *Nat. Commun.* **7**, 10807 (2016).
